# Relative transmissibility of shigellosis among male and female individuals: a modeling study in Hubei Province, China

**DOI:** 10.1186/s40249-020-00654-x

**Published:** 2020-04-17

**Authors:** Ze-Yu Zhao, Qi Chen, Bin Zhao, Mikah Ngwanguong Hannah, Ning Wang, Yu-Xin Wang, Xian-Fa Xuan, Jia Rui, Mei-Jie Chu, Shan-Shan Yu, Yao Wang, Xing-Chun Liu, Ran An, Li-Li Pan, Yi-Chen Chiang, Yan-Hua Su, Ben-Hua Zhao, Tian-Mu Chen

**Affiliations:** 1 State Key Laboratory of Molecular Vaccinology and Molecular Diagnostics, School of Public Health, Xiamen University, Xiamen City, Fujian Province, 4221-117 South Xiang’an Road, Xiang’an District, Xiamen, Fujian Province People’s Republic of China; 2 Hubei Provincial Center for Disease Control and Prevention, Wuhan City, Hubei Province People’s Republic of China; 3 Laboratory Department, Xiang’an Hospital of Xiamen University, State Key Laboratory of Molecular Vaccinology and Molecular Diagnosis, Xiamen, Fujian People’s Republic of China; 4 Medical College, Xiamen University, Xiamen City, Fujian Province People’s Republic of China; 5 Respiratory Department, Shanghai General Hospital, Shanghai, People’s Republic of China; 6 Department of Nephrology, The Second Affiliated Hospital of Xiamen Medical College, Xiamen, Fujian People’s Republic of China

**Keywords:** Shigellosis, Transmissibility, Mathematical model, Gender

## Abstract

**Background:**

Developing countries exhibit a high disease burden from shigellosis. Owing to the different incidences in males and females, this study aims to analyze the features involved in the transmission of shigellosis among male (subscript *m*) and female (subscript *f*) individuals using a newly developed sex-based model.

**Methods:**

The data of reported shigellosis cases were collected from the China Information System for Disease Control and Prevention in Hubei Province from 2005 to 2017. A sex-based Susceptible–Exposed–Infectious/Asymptomatic–Recovered (SEIAR) model was applied to explore the dataset, and a sex-age-based SEIAR model was applied in 2010 to explore the sex- and age-specific transmissions.

**Results:**

From 2005 to 2017, 130 770 shigellosis cases (including 73 981 male and 56 789 female cases) were reported in Hubei Province. The SEIAR model exhibited a significant fitting effect with the shigellosis data (*P* <  0.001). The median values of the shigellosis transmission were 2.3225 × 10^8^ for *SAR*_*mm*_ (secondary attack rate from male to male), 2.5729 × 10^8^ for *SAR*_*mf*_, 2.7630 × 10^-8^ for *SAR*_*fm*_, and 2.1061 × 10^-8^ for *SAR*_*ff*_. The top five mean values of the transmission relative rate in 2010 (where the subscript 1 was defined as male and age ≤ 5 years, 2 was male and age 6 to 59 years, 3 was male and age ≥ 60 years, 4 was female and age ≤ 5 years, 5 was female and age 6 to 59 years, and 6 was male and age ≥ 60 years) were 5.76 × 10^-8^ for *β*_61_, 5.32 × 10^-8^ for *β*_31_, 4.01 × 10^-8^ for *β*_34_, 7.52 × 10^-9^ for *β*_62_, and 6.04 × 10^-9^ for *β*_64_.

**Conclusions:**

The transmissibility of shigellosis differed among male and female individuals. The transmissibility between the genders was higher than that within the genders, particularly female-to-male transmission. The most important route in children (age ≤ 5 years) was transmission from the elderly (age ≥ 60 years). Therefore, the greatest interventions should be applied in females and the elderly.

## Background

Shigellosis, also known as bacillary dysentery, is an infectious disease caused by the genus *Shigella* spp., which frequently occurs in summer and autumn. *Shigella flexneri* is the main cause of endemic diarrhea in low and middle income countries, and lays a heavy burden on these countries, particularly in children aged 1 to 4 years old [1]. According to the Chinese Center for Disease Control and Prevention (China CDC), approximately 150 000 to 450 000 cases were reported annually within the period 2005 to 2014 [2]. Although there have been an improvement in the quality of water and sanitation, shigellosis remains a major public health problem in several developing countries, including China [3, 4].

Bacillary dysentery is an infectious intestinal disease that can be transmitted via the consumption of contaminated food or water [5]. Humans are the only natural host for *Shigella* spp.. In recent years, numerous reports have demonstrated that the incidence of shigellosis within males is higher than that within females [6–8]. The incidence of shigellosis, a water/food born disease, is directly related to the hygiene behaviours such as regular hand washing [9]. A study has indicated that the sanitary state in females is always higher than that in males [10]. Does this mean that the transmission features differ between male and female? A study has reported that shigellosis primarily occurs from person-to-person [1]. Thus, the water/food-to-person route has been interrupted. Moreover, many studies have indicated different incidences in individuals of various ages [1, 8, 11]. In this study, we aimed to explore the interpersonal transmission further.

In model studies of shigellosis, the distribution of time and space has been a greater focus than population-based research [12–16]. A study demonstrated that the Susceptible–Exposed–Infectious/Asymptomatic–Recovered–Water/Food (SEIARW) model exhibited a significant fitting effect with outbreak data in a school [17]. However, it did not estimate the transmissibility of bacillary dysentery between males and females. Considering that water makes less of a contribution in the transmission, a sex-based Susceptible–Exposed–Infectious/Asymptomatic–Recovered (SEIAR) model was applied to explore the dataset from Hubei Province. The secondary attack rate (*SAR*), which is defined as the probability of an infected person infecting a susceptible person during his or her entire infectious period, was adopted to assess the relative transmissibility of shigellosis between males and females. In this study, shigellosis cases reported in Hubei Province, China, were collected. The SEIAR model was applied to fit the data, calculate the related index, and determine the transmissibility of shigellosis between males and females. With the aim of exploring the transmission features in different gender and age groups, the SEIAR model was adopted to fit the data of shigellosis cases reported from 2005 to 2017 in Hubei Province, China.

## Methods

### Study design

**Fig. 1:**
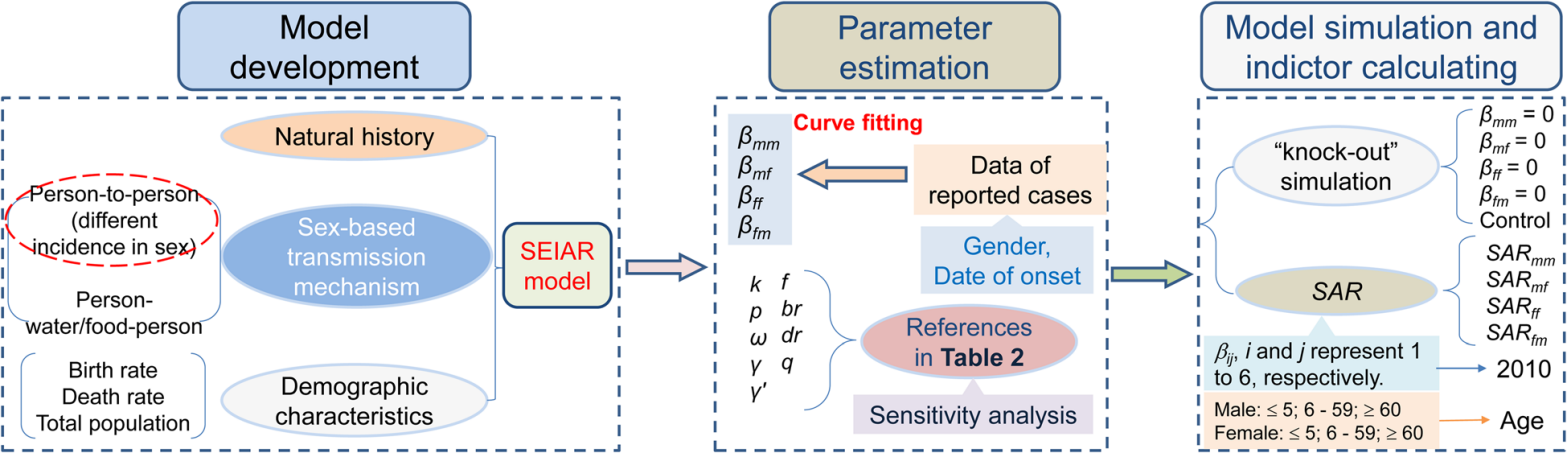
Framework for model development. SEIAR: Susceptible–Exposed–Infectious/Asymptomatic–Recovered; *SAR*: secondary attack rate

A mathematical study was implemented using a sex- and age-based model to analyze the transmission characteristics of reported shigellosis cases in Hubei Province, China, from 2005 to 2017. In this study, we divided the research process into three parts (Fig. 1). First, we developed the model according to the natural history and transmission mechanism in different genders. Second, we acquired the model parameters by reference and curve fitting. Finally, we adopted indicators to estimate the transmissibility in different genders and to explore the transmission features in different age groups further.

### Data collection

The dataset of the shigellosis cases was collected from the China Information System for Disease Control and Prevention in Hubei Province from 2005 to 2017. The dataset included gender, age, occupation, address, date of onset, and date of diagnosis. In this study, people were divided into two groups according to gender. The information of the population, such as the birth rate, death rate and total population were obtained from the Hubei Statistical Yearbook.

### Shigellosis model between different genders

**Fig. 2:**
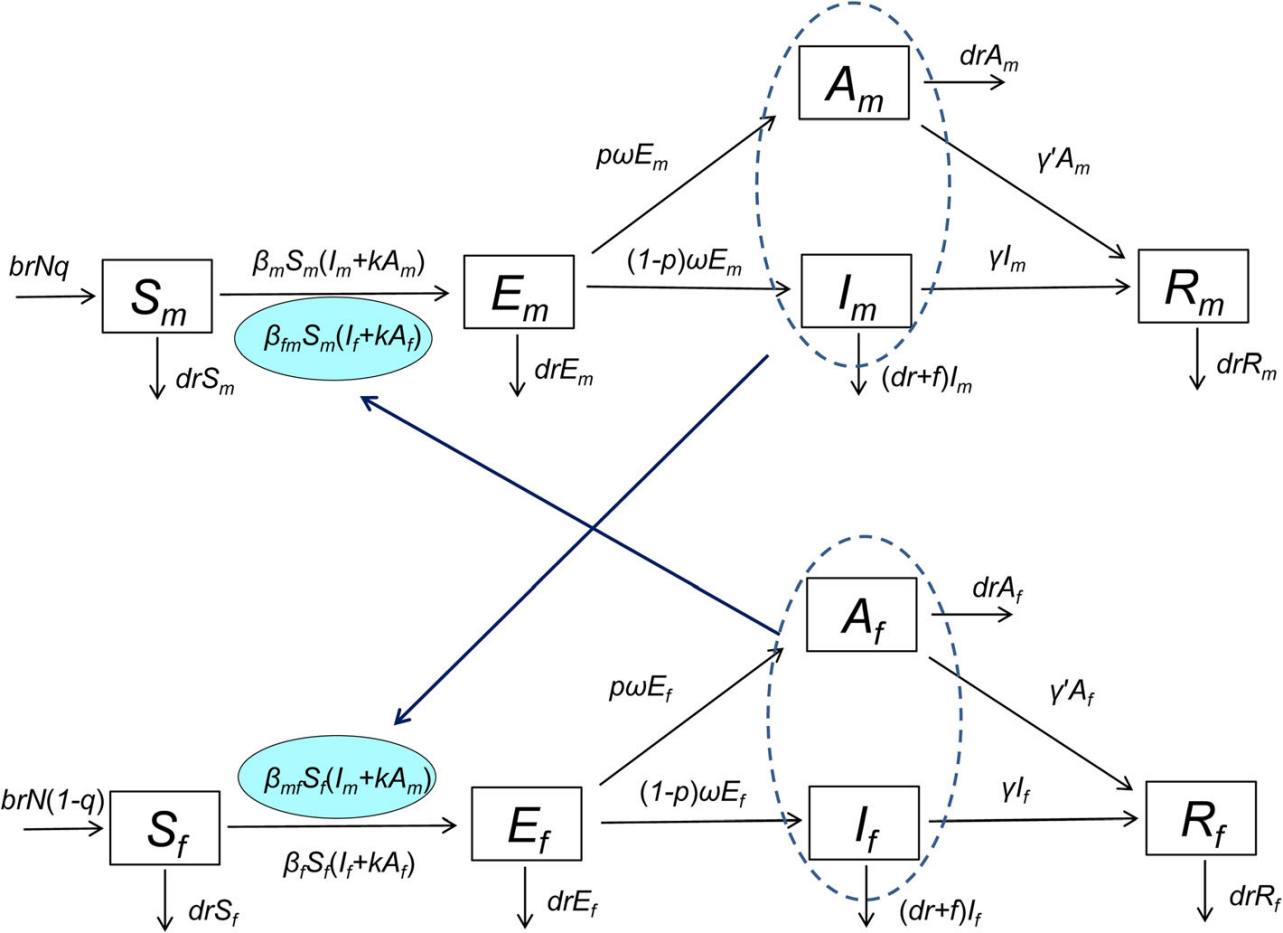
Flowchart of transmission Susceptible–Exposed–Infectious/Asymptomatic–Recovered model of shigellosis in different genders. Male: subscript *m*; Female: subscript *f*

The SEIAR model was developed according to the natural history of shigellosis among male and female individuals (Fig. 2). We used the subscripts *m* to represent male and *f* to represent female. The pattern followed by the model was person to person, which consisted of susceptible (*S*_*m*_, *S*_*f*_), exposed (*E*_*m*_, *E*_*f*_), symptomatic (*I*_*m*_, *I*_*f*_), asymptomatic (*A*_*m*_, *A*_*f*_) and recovered (*R*_*m*_, *R*_*f*_) individuals. Definitions of the epidemiological classes are summarized in Table 1. In the model, we assumed that:

- Susceptible individuals of different genders become infected by contact with infected/asymptomatic people.
- The relative rate of transmission among male and female individuals is *β*_*mm*_ and *β*_*ff*_, respectively.
- The relative rate of transmission from male to female is *β*_*mf*_ and from female to male is *β*_*fm*_.

**Table 1:** Variables in the intersex transmission SEIAR model

Variable	Description	Unit
*S*_*m*_	Male susceptible individuals density	Individuals·km^-2^
*S*_*f*_	Female susceptible individuals density	Individuals·km^-2^
*E*_*m*_	Male exposed individuals density	Individuals·km^-2^
*E*_*f*_	Female exposed individuals density	Individuals·km^-2^
*I*_*m*_	Male infectious individuals density	Individuals·km^-2^
*I*_*f*_	Female infectious individuals density	Individuals·km^-2^
*A*_*m*_	Male asymptomatic individuals density	Individuals·km^-2^
*A*_*f*_	Female asymptomatic individuals density	Individuals·km^-2^
*R*_*m*_	Male recovered individuals density	Individuals·km^-2^
*R*_*f*_	Female recovered individuals density	Individuals·km^-2^
*N*	Total number of population density	Individuals·km^-2^

*SEIAR* Susceptible–Exposed–Infectious/Asymptomatic–Recovered

Moreover, we assumed that in both male and female:

- The disease does not spread vertically, and individuals born in various groups are all susceptible. The natural birth rate is *br* and the natural mortality rate is *dr*.
- According to a new review [1], the transmission of shigellosis mainly occurs from person-to-person. Meanwhile, our pilot study indicated a minor contribution of water/food (Additional file 1). Therefore, we assumed that the water/food to person transmission route had been cut off.
- The (*1-p*) *E* (0 ≤ *p* ≤ 1) number of exposed individuals will change to infected person *I* following an incubation period, while a further *pE* number of exposed individuals will become asymptomatic person *A* following a latent period (the period during which the exposed individuals become an asymptomatic person).
- The removal speed from *I* and *A* is positively proportional to the number of people in both groups, and the proportional coefficients are *γ* and *γ’*, respectively, whereas 1/*γ* and 1/*γ’* are the infectious period of *I* and *A*.
- The infected person will die as a result of the disease and the case fatality rate is *f*.

The model is expressed as follows:

$$ \frac{d{S}_m}{dt}= brNq-{\beta}_{mm}{S}_m\left({I}_m+k{A}_m\right)-{\beta}_{fm}{S}_m\left({I}_f+k{A}_f\right)- dr{S}_m $$

$$ \frac{d{E}_m}{dt}={\beta}_{mm}{S}_m\left({I}_m+k{A}_m\right)+{\beta}_{fm}{S}_m\left({I}_f+k{A}_f\right)-\omega {E}_m- dr{E}_m $$

$$ \frac{d{I}_m}{dt}=\left(1-p\right)\omega {E}_m-\gamma {I}_m-\left( dr{I}_m+f{I}_m\right) $$

$$ \frac{d{A}_m}{dt}= p\omega {E}_m-{\gamma}^{\prime }{A}_m- dr{A}_m $$

$$ \frac{d{R}_m}{dt}={\gamma I}_m+{\gamma}^{\prime }{A}_m- dr{R}_m $$

$$ \frac{d{S}_f}{dt}= brN\left(1-q\right)-{\beta}_{ff}{S}_f\left({I}_f+k{A}_f\right)-{\beta}_{mf}{S}_f\left({I}_m+k{A}_m\right)- dr{S}_f $$

$$ \frac{d{E}_f}{dt}={\beta}_{ff}{S}_f\left({I}_f+k{A}_f\right)+{\beta}_{mf}{S}_f\left({I}_m+k{A}_m\right)-\omega {E}_f- dr{E}_f $$

$$ \frac{d{I}_f}{dt}=\left(1-p\right)\omega {E}_f-\gamma {I}_f-\left( dr{I}_f+f{I}_f\right) $$

$$ \frac{d{A}_f}{dt}= p\omega {E}_f-{\gamma}^{\prime }{A}_f- dr{A}_f $$

$$ \frac{d{R}_f}{dt}={\gamma I}_f+{\gamma}^{\prime }{A}_f- dr{R}_f $$

$$ N={S}_m+{E}_m+{I}_m+{A}_m+{R}_m+{S}_f+{E}_f+{I}_f+{A}_f+{R}_f $$

The left side of the equation indicates the instantaneous rate of change of *S*, *E*, *I*, *A* and *R* at time *t*. In the model, the *SAR* was calculated as follows:

$$ {SAR}_{mm}={\beta}_{mm}/\gamma $$

$$ {SAR}_{mf}={\beta}_{mf}/\gamma $$

$$ {SAR}_{fm}={\beta}_{fm}/\gamma $$

$$ {SAR}_{ff}={\beta}_{ff}/\gamma $$

Considering that the transmissibility could relate to different ages (we considered three age groups based on the age distribution of the reported shigellosis incidences in the province), we divided individuals into six groups. The subscript 1 was defined as male and age ≤ 5 years, 2 was male and age 6 to 59 years, 3 was male and age ≥ 60 years, 4 was female and age ≤ 5 years, 5 was female and age 6 to 59 years, and 6 was male and age ≥ 60 years. Thereafter, we constructed a sex–age-based SEIAR model. We calculated the ratios *x*, *y*, and *z* (from the results of sex-based SEIAR model) in four transmission routes of the different genders to increase the reliability of the estimated parameters. We set *β*_*ff*_ as *β*_0_ and

$$ {\beta}_{mm}=x\ast {\beta}_0,{\beta}_{fm}=y\ast {\beta}_0,{\beta}_{mf}=z\ast {\beta}_0. $$

**Fig. 3:**
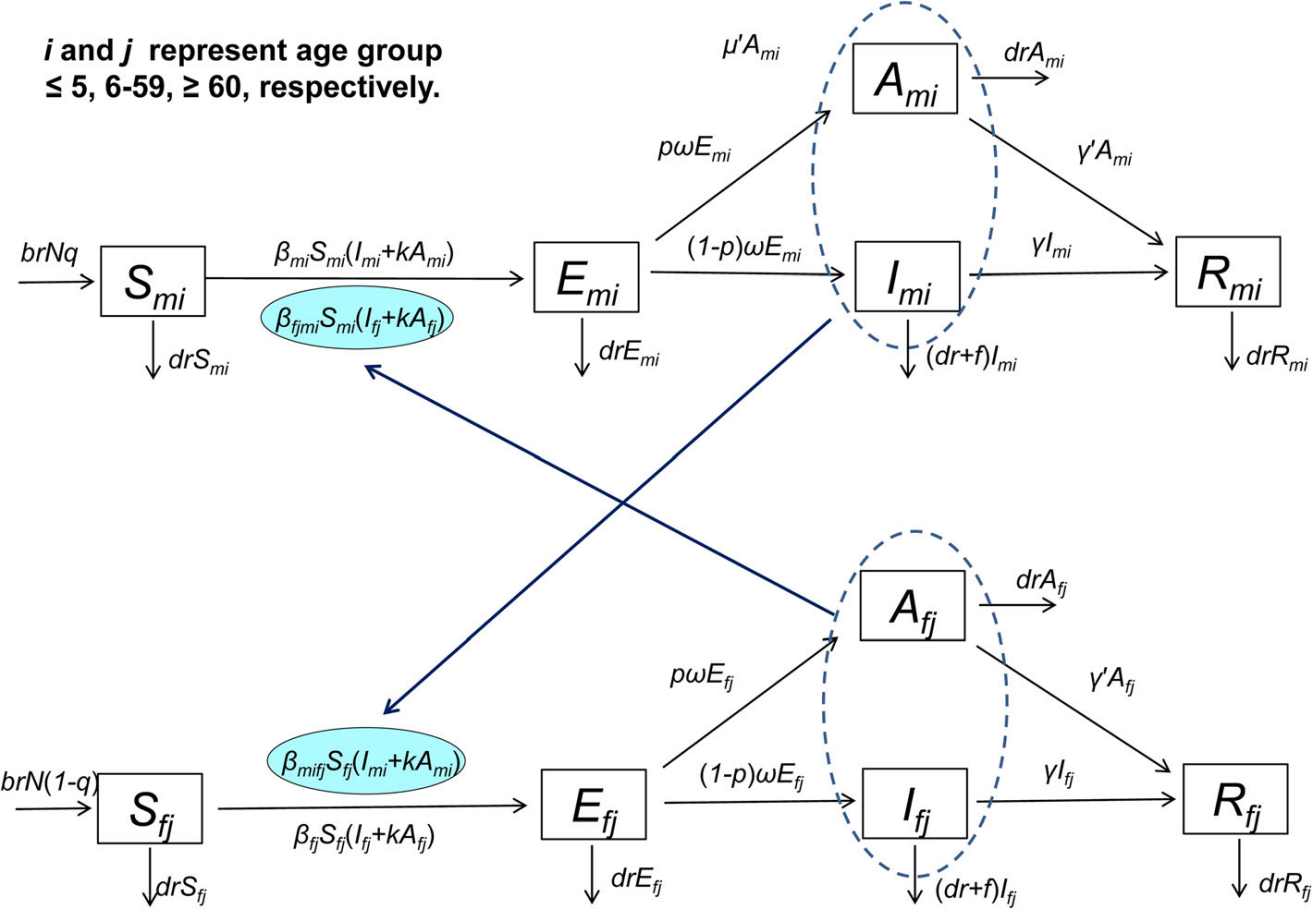
Flowchart of transmission Susceptible–Exposed–Infectious/Asymptomatic–Recovered model of shigellosis in different age and gender groups. Male: subscript *m*; Female: subscript *f*; *i* and *j* represent age ≤ 5, 6–59, ≥ 60, respectively

**Fig. 4:**
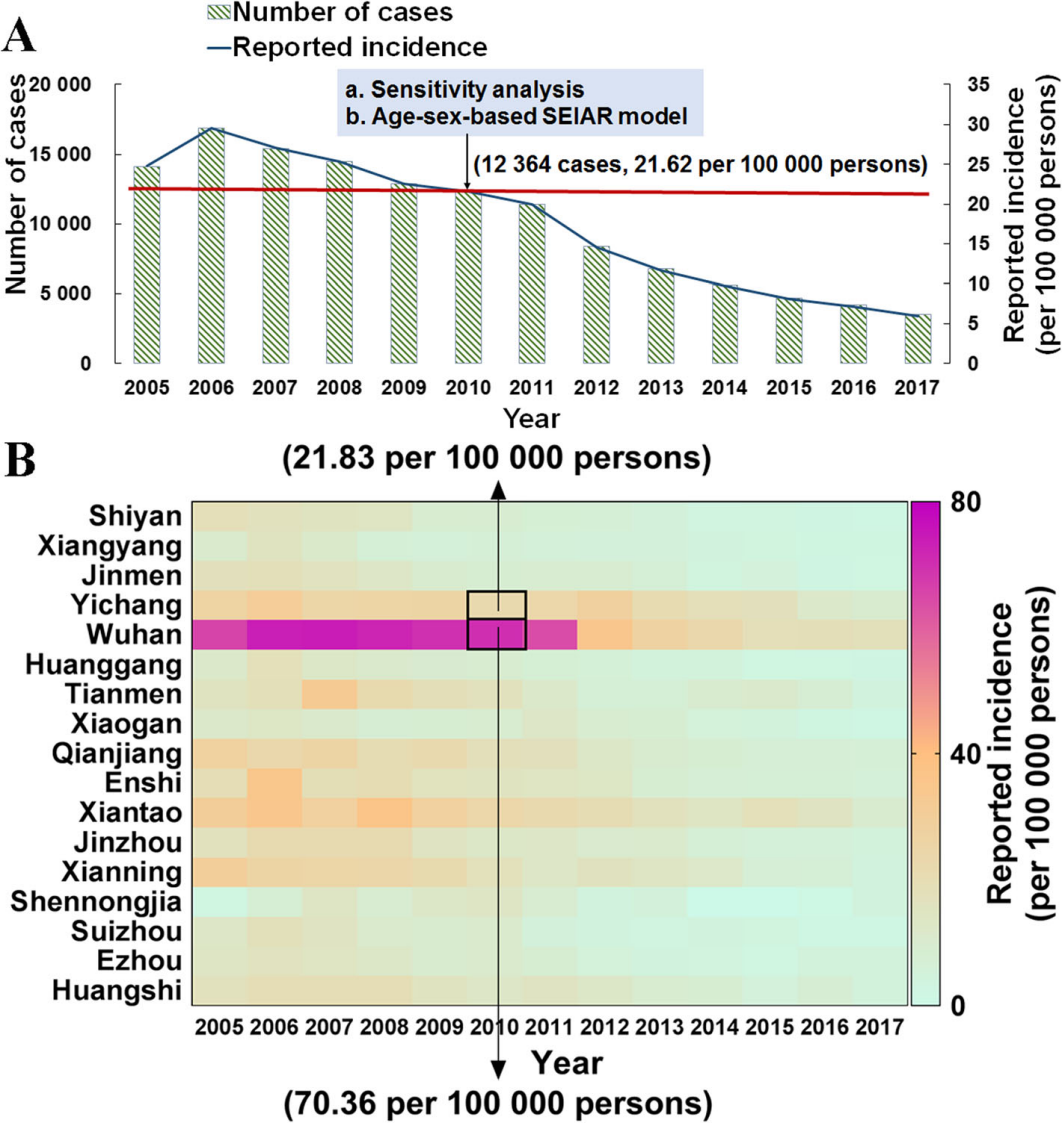
The incidence of Hubei Province and 17 cities of it from 2005 to 2017. **a**: Hubei Province; **b**: 17 cities of Hubei Province

The framework is presented in Fig. 3 and its equation is provided in Additional file 2. According to the reported incidence of shigellosis from 2005 to 2017 in Hubei Province, we selected the year 2010 to quantify the transmissibility in the different sex and age groups (Fig. 4a). Meanwhile, we compared Wuhan City with Yichang City based on the different incidence in both cities of Hubei Province in 2010 (Fig. 4b).

### Parameter estimation

According to the epidemiological characteristics of shigellosis and our previous study [17], we set *k* and *γ’* as 0.3125 and 0.0286, respectively. The proportions of asymptomatic individuals were reported to range from 0.0037 to 0.2700 [18–20]. We set *p* = 0.1 in the SEIAR model. The incubation of shigellosis was reported to range from 1 to 3 days [21–23]. Therefore, we set *ω* as 0.3333 to 1.000. The symptoms generally last for 1 week, but certain people may experience symptoms for several weeks [24, 25]. We assumed the course of the disease was up to 3 weeks. Therefore, we set *γ* as 0.0477 to 0.1428. The fatality rate of the disease reported in a study decreased from 0.00088 to 0.00031 from 1991 to 2000 [26]. Considering that the fatality rate of shigellosis is extremely low [27], we set *f* = 0. The values of *β*_*mm*_, *β*_*ff*_, *β*_*mf*_ and *β*_*fm*_ were generated by curve fitting using the SEIAR model and the reported shigellosis data. The definitions, ranges and sources of the parameters are displayed in Table 2.

**Table 2:** Parameter description and values of SEIAR model

Parameter	Description	Unit	Value	Range	Method
*β*_*mm*_	Transmission relative rate among male individuals	Individuals^-1^·days^-1^	see text	≥ 0	Curve fitting
*β*_*ff*_	Transmission relative rate among female individuals	Individuals^-1^·days^-1^	see text	≥ 0	Curve fitting
*β*_*fm*_	Transmission relative rate from female to male	Individuals^-1^·days^-1^	see text	≥ 0	Curve fitting
*β*_*mf*_	Transmission relative rate from male to female	Individuals^-1^·days^-1^	see text	≥ 0	Curve fitting
*k*	Relative transmissibility rate of asymptomatic to symptomatic individuals	1	0.3125	0–1	References [17]
*p*	Proportion of the asymptomatic	1	0.1	0.0037–0.2700	References [18–20]
*ω*	Incubation relative rate	days^-1^	1	0.3333–1.000	References [21–23]
*γ*	Recovery rate of the infectious	days^-1^	0.0741	0.0477–0.1428	References [24, 25]
*γ’*	Recovery rate of the asymptomatic	days^-1^	0.0286	0–0.0357	References [17]
*f*	Fatality of the disease	1	0	0.0003–0.0009	References [26, 27]
*br*	Birth rate of the population	1	-	0.00002384–0.00003452	Hubei Statistical Yearbook
*dr*	Death rate of the population	1	-	0.00001562–0.00001918	Hubei Statistical Yearbook
*q*	Proportion of male	1	-	0.5078–0.5186	Hubei Statistical Yearbook

Note: - : Not applicable;

*SEIAR* Susceptible–Exposed–Infectious/Asymptomatic–Recovered

We performed a “knock-out” simulation to explore the roles of the different *β* values. The theory of the “knock-out” simulation was come from originates from the gene “knock-out” technique (an experimental technique used in genetics in which a normal gene is replaced by a defective gene either at the exact same chromosomal site—hence, the normal gene is ‘knocked out’ by the defective gene—as occurs with the yeast genome, or the deoxyribonucleic acid is inserted at random sites, as occurs in mammalian cells) [28]. In the model, we always estimated the contribution of one parameter by setting it to 0 to calculate the decreasing number of cases or total attack rate. For example, the contribution of the parameter *β*_*fm*_ simulated by the model was the decreasing number of cases when we set it to 0.

Therefore, “knock-out” simulation (interrupting the different shigellosis transmission routes among males and females) was performed in five scenarios in our study: A) *β*_*mm*_ = 0; B) *β*_*mf*_ = 0; C) *β*_*ff*_ = 0; D) *β*_*fm*_ = 0; and E) control (no intervention).

### Simulation method and statistical analysis

**Fig. 5:**
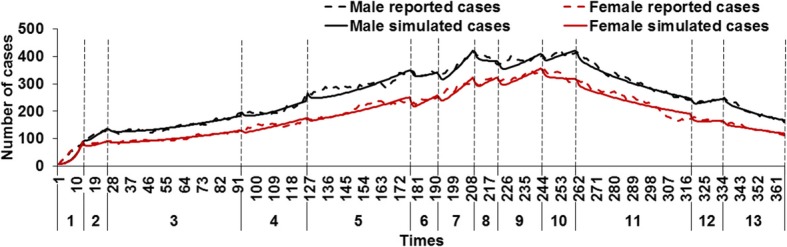
The division times according to number of cases reported per day of Hubei Province in 2010

Berkeley Madonna 8.3.18 (developed by Robert Macey and George Oster of the University of California at Berkeley; Copyright©1993–2001 Robert I. Macey & George F. Oster, University of California, Berkeley, CA) was employed for the model simulation. The simulation methods were as previously described [17, 29–32]. According to our previous published studies [33, 34], we assumed that heterogeneity of the transmissibility existed during an ascending trend and a descending trend. The annual data were therefore divided into numerous parts and the simulated time step was a day; for example, the data of 2010 were divided into 13 parts (Fig. 5). The Runge-Kutta method of order 4 with the tolerance set at 0.001 was used for model building. Berkeley Madonna minimized the root mean square during the curve fitting process. Microsoft Office Excel 2016 (Microsoft, Redmond, WA, USA) and GraphPad Prism 7.0 (GraphPad Software, La Jolla, CA) were employed for the figure development and data analysis. Moreover, SPSS 21.0 (IBM Corp, Armonk, NY, USA) was used to calculate the coefficient of determination (*R*^*2*^) by curve fitting, which was adopted to judge the model goodness of fit.

### Sensitivity analysis

Because nine parameters, namely *k*, *ω*, *γ*, *γ’*, *p*, *br*, *dr*, *f* and *q*, were obtained from references and the Hubei Statistical Yearbook, uncertainty existed influence in the model. In our model, the nine parameters were split into 1000 values, as indicated in Table 2. Considering that the simulated model method was the same in each year, we performed sensitivity analysis in 2010 (a middle reported incidence and case in Fig. 4a).

## Results

### Epidemiological characteristics of reported shigellosis cases

**Fig. 6:**
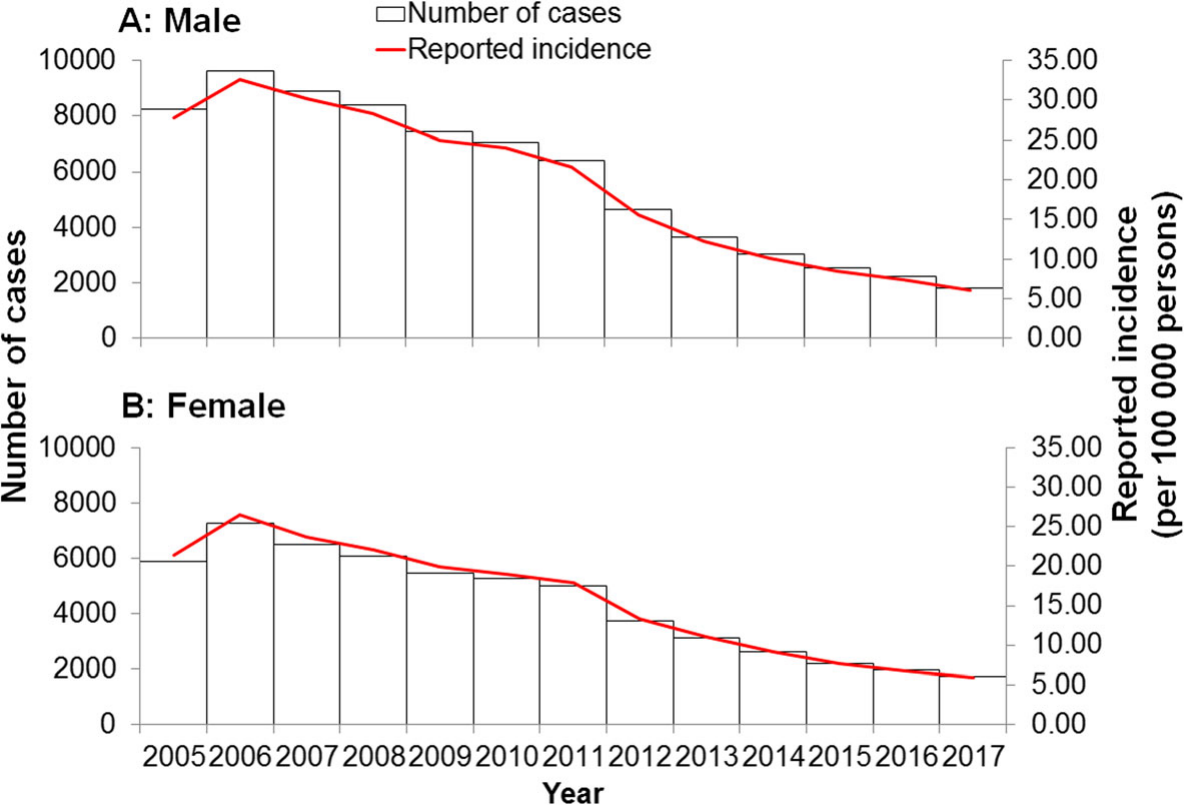
Reported cases and incidence of shigellosis in different genders from 2005 to 2017 in Hubei province. **a**: Male; **b**: Female

From 2005 to 2017, a total of 130 770 shigellosis cases (including 73 981 male cases and 56 789 female cases) were reported in Hubei Province (Fig. 6). The median of incidences reported annually was 21.68 per 100 000 persons (range: 6.10 to 32.63 per 100 000 persons) in males and 17.91 per 100 000 persons (range: 5.87 to 26.51 per 100 000 persons) in females. This demonstrated that, the number of cases and reported incidences in males and females had decreased significantly (male trend: *χ*^*2*^ = 11.268, *P* = 0.001, Female trend: *χ*^*2*^ = 11.144, *P* = 0.001).

### Curve fitting results

**Fig. 7:**
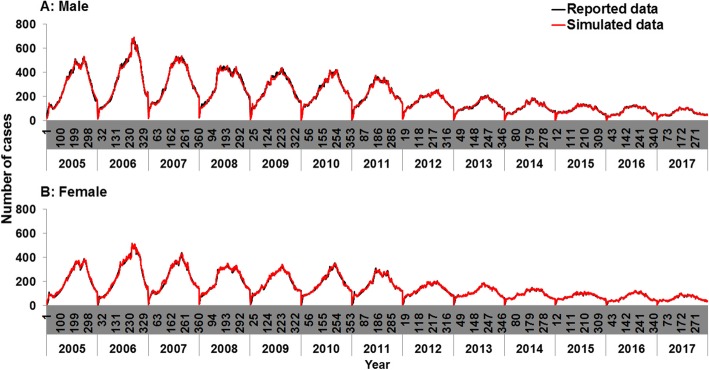
Curve fitting of Model to reported data in different genders from 2005 to 2017 in Hubei. **a**: Male; **b**: Female

**Fig. 8:**
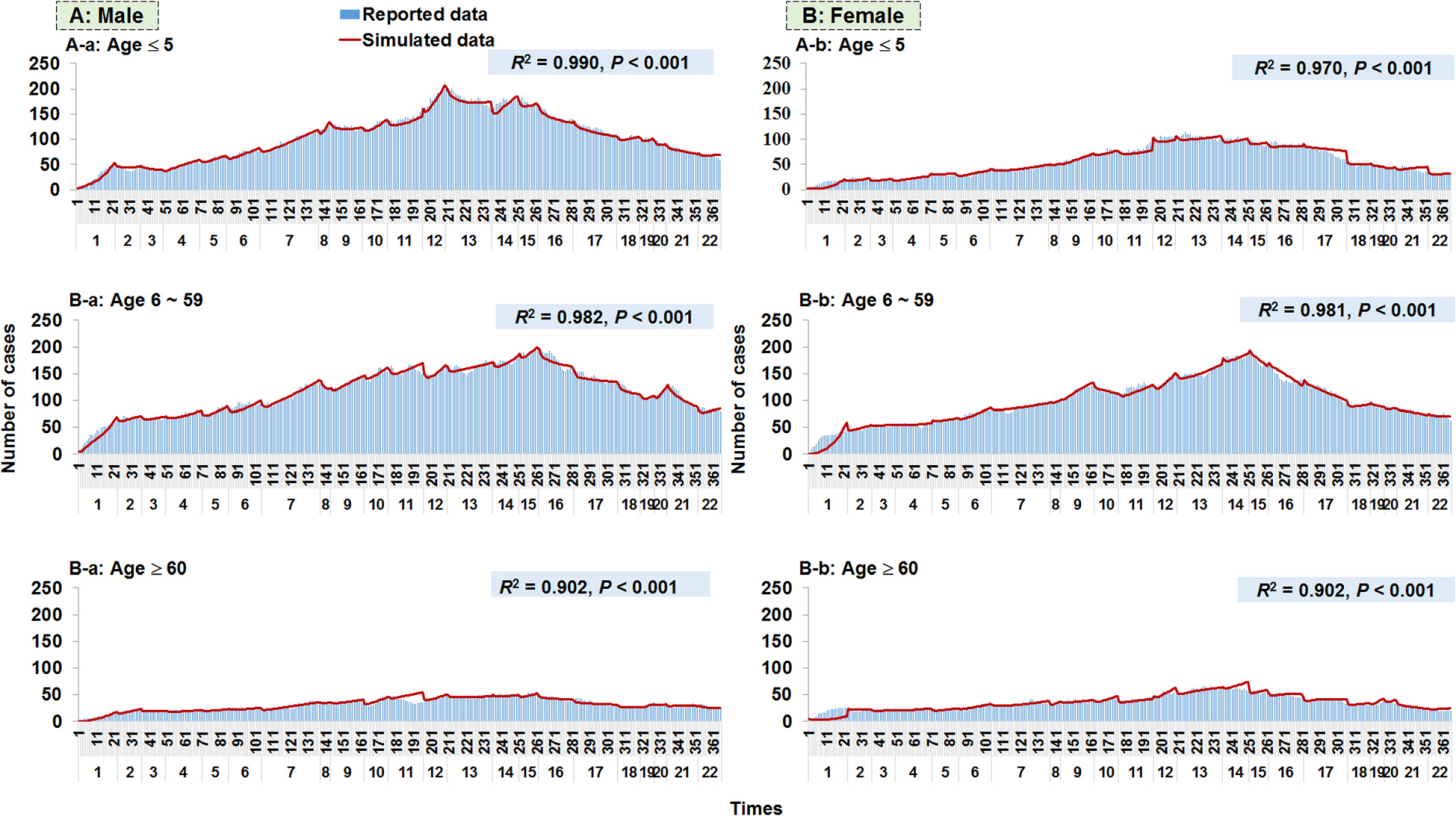
Curve fitting of Model to reported data in different age and gender groups in 2010. A: Male; A-a: Age ≤ 5; A-b: Age 6–59; A-c: Age ≥ 60; B: Female; B-a: Age ≤ 5; B-b: Age 6–59; B-c: Age ≥ 60

**Fig. 9:**
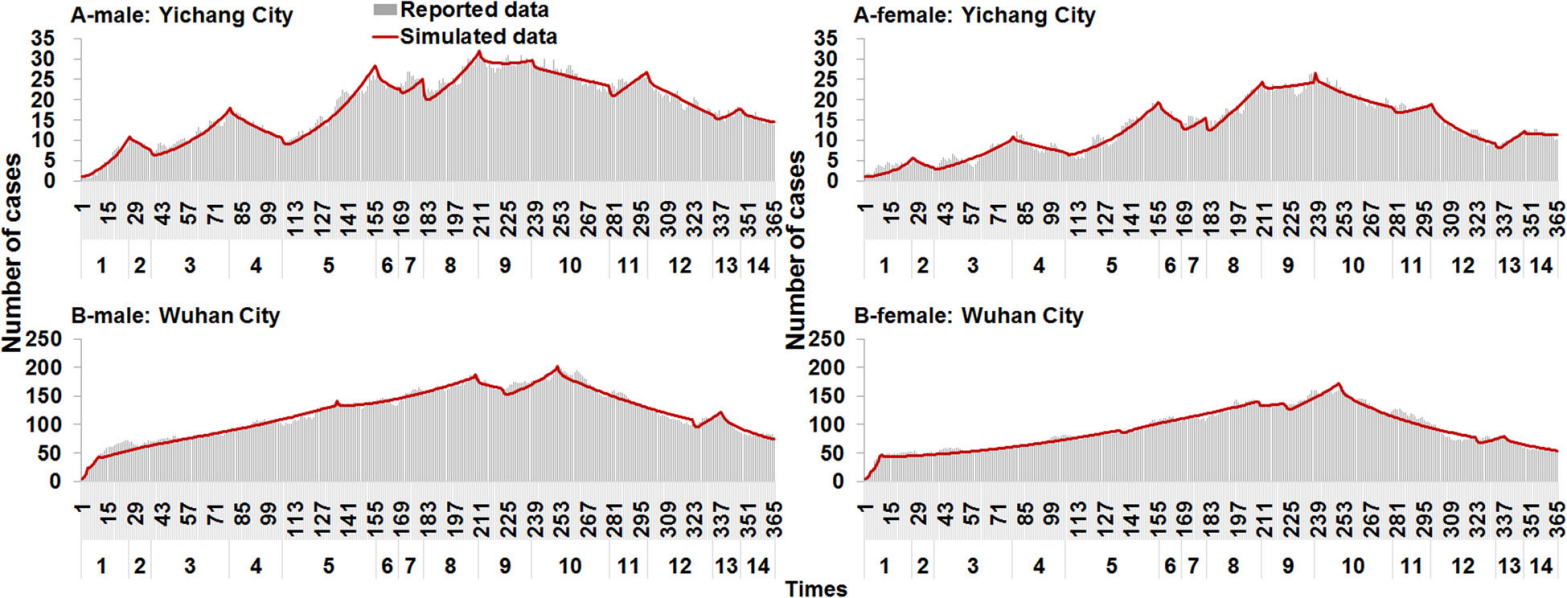
Curve fitting of Model to reported data in Wuhan City and Yichang City in 2010. A-male: Yichang City; B-male: Wuhan City; **a**-female: Yichang City; **b**-female: Wuhan City

The results of the curve fitting indicated that the SEIAR model fitted the data effectively (Fig. 7). The *R*^*2*^ values of the SEIAR model for the different genders each year are presented in Table 3. In 2010, the reported data of all individual groups exhibited a significant fitting effect with simulated data in Hubei Province (Fig. 8), Wuhan City, and Yichang City (Fig. 9).

**Table 3:** *R*^*2*^ of model and reported cases in different genders from 2005 to 2017 in Hubei Province, China

Year	Male		Female	
	*R*^*2*^	*P*	*R*^*2*^	*P*
2005	0.989	< 0.001	0.991	< 0.001
2006	0.995	< 0.001	0.992	< 0.001
2007	0.992	< 0.001	0.987	< 0.001
2008	0.984	< 0.001	0.986	< 0.001
2009	0.982	< 0.001	0.984	< 0.001
2010	0.989	< 0.001	0.982	< 0.001
2011	0.985	< 0.001	0.982	< 0.001
2012	0.989	< 0.001	0.979	< 0.001
2013	0.977	< 0.001	0.983	< 0.001
2014	0.986	< 0.001	0.983	< 0.001
2015	0.977	< 0.001	0.965	< 0.001
2016	0.985	< 0.001	0.988	< 0.001
2017	0.986	< 0.001	0.978	< 0.001

### Transmissibility of shigellosis in different genders

**Fig. 10:**
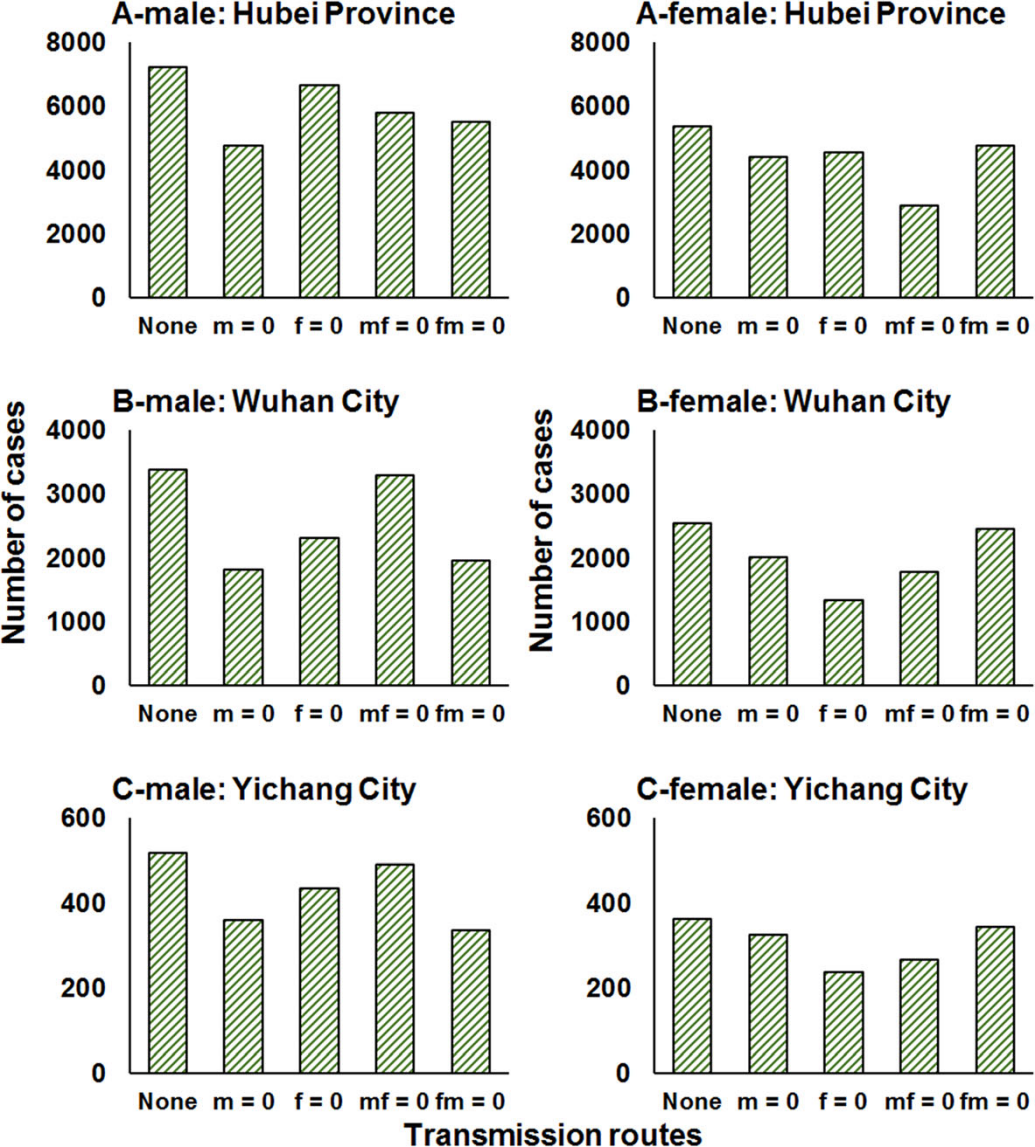
The “knock-out” simulation of Hubei Province, Wuhan and Yichang City in 2010. A-male: Hubei Province; A-female: Hubei Province; B-male: Wuhan City; B-female: Wuhan City; C-male: Yichang City; C-female: Yichang City; m = 0: interrupt transmission among male; f = 0: interrupt transmission among female; mf = 0: interrupt transmission from male to female; fm = 0: interrupt transmission from female to male; None: control

According to Fig. 10, the results of the “knock-out” simulation demonstrated that the number of cases in the different genders using the parameters *β*_*mm*_ = 0, *β*_*ff*_ = 0, *β*_*mf*_ = 0 and *β*_*fm*_ = 0 were lower than that in the control group. When *β*_*fm*_ = 0, the number of cases decreased the most in the different genders.

**Fig. 11:**
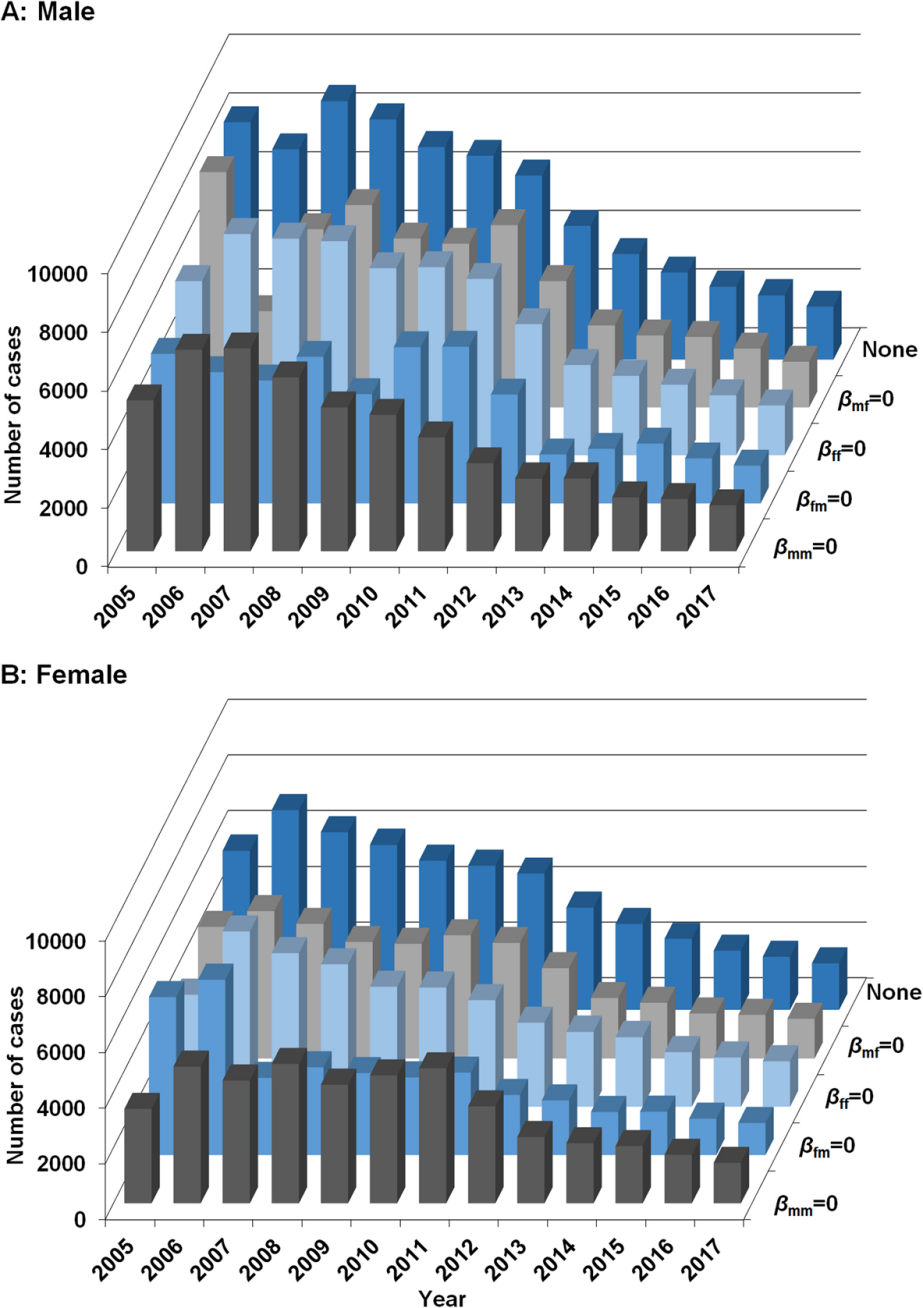
The results to simulate the contribution of *β* during the transmission in different genders. **a**: Male; **b**: Female; *β*_*mm*_ = 0, interrupt transmission among male; *β*_*ff*_ = 0, interrupt transmission among female; *β*_*fm*_ = 0, interrupt transmission from female to male; *β*_*mf*_ = 0, interrupt transmission from male to female; None: control

In 2010, a total of 12 340 cases were reported in Hubei Province (873 cases in Yichang City and 5 899 cases in Wuhan City). The “knock-out” simulation demonstrated similar results of the contribution in four transmission routes between Wuhan and Yichang City, but different results from Hubei Province (Fig. 11).

**Fig. 12:**
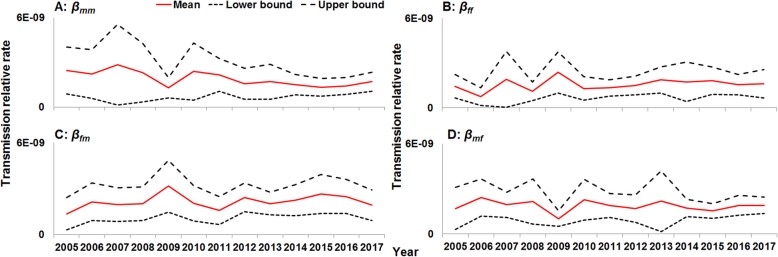
The parameter of *β*_*mm*_, *β*_*ff*_, *β*_*mf*_ and *β*_*fm*_ during the transmission from 2005 to 2017 in Hubei. **a**: *β*_*mm*_, transmission relative rate among male; **b**: *β*_*ff*_, transmission relative rate among female; **c**: *β*_*mf*_, transmission relative rate from male to female; **d**: *β*_*fm*_, transmission relative rate from female to male

Fig. 12 presents the difference between the mean and 95% confidence interval (*CI*) from 2005 to 2017 when using *β*_*mm*_, *β*_*ff*_, *β*_*mf*_ and *β*_*fm*_. The mean value was 1.9240 × 10^-9^ (95% *CI*: 1.6621 × 10^-9^ to 6.6121 × 10^-9^) when using *β*_*mm*_, 1.5645 × 10^-9^ (95% *CI*: 1.3521 × 10^-9^ to 1.7769 × 10^9^) when using *β*_*ff*_, 2.1572 × 10^-9^ (95% *CI*: 1.9159 × 10^-9^to 2.3986 × 10^-9^) when using *β*_*fm*,_ and 1.8750 × 10^-9^ (95% *CI*: 1.6846 × 10^-9^ to 2.0654 × 10^-9^) when using *β*_*mf*_.

**Fig. 13:**
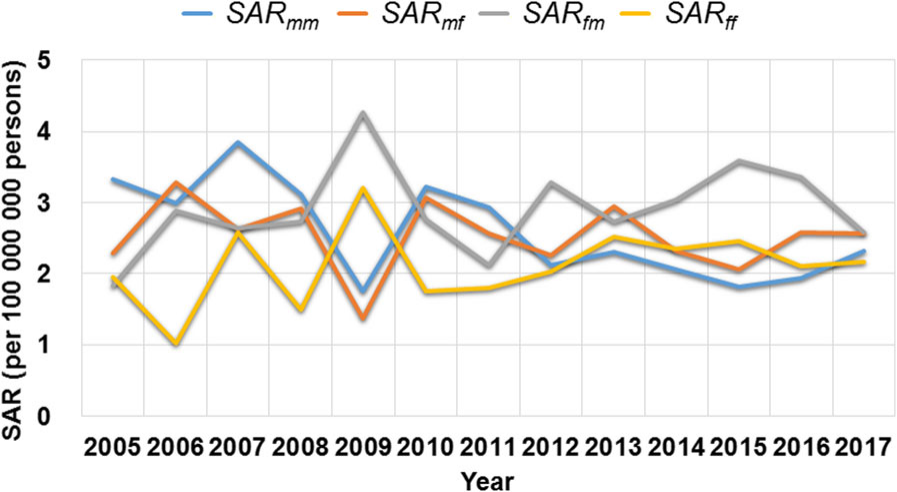
The *SAR*_*mm*_*, SAR*_*mf*_*, SAR*_*fm*_ and *SAR*_*ff*_ estimated by model from 2005 to 2017 in Hubei. *SAR*: secondary attack rate; subscript *mm*, among male; *mf*, from male to female; *fm*, from female to male; *ff*, among female

The results of the *SAR* from 2005 to 2017 are presented in Fig. 13. The median value of *SAR*_*mm*_ was 2.32 (Range: 1.76–3.86) per 100 000 000 persons. The median value of *SAR*_*mf*_ was 2.57 (Range: 1.38–3.28) per 100 000 000 persons. The median value of *SAR*_*fm*_ was 2.76 (Range: 1.84–4.26) per 100 000 000 persons. The median value of *SAR*_*ff*_ was 2.11 (Range: 1.02–3.21) per 100 000 000 persons.

### Transmissibility in different sex–age groups

**Fig. 14:**
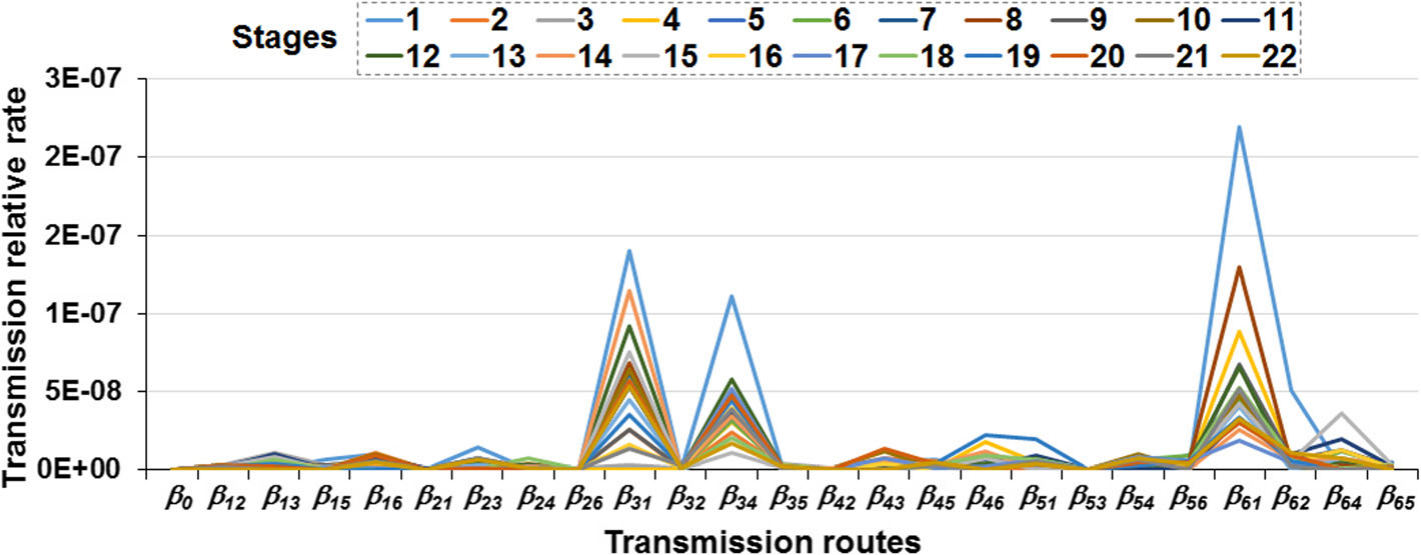
The transmission relative rate in different age and gender groups in 2010. *β*_0_: transmission relative rate within female; *β*_*ij*_ refers to transmission relative rate of gender and age group from *i* to *j*, *i* and *j* represent subscript 1 to 6, subscript 1 was defined as male and ≤ 5 years old, 2 was male and between 6 to 59 years old, 3 was male and ≥ 60 years old, 4 was female and ≤ 5 years old, 5 was female and between 6 to 59 years old, and 6 was female and ≥ 60 years old; The data of 2010 were divided into 22 stages based on the following simulated periods, 1: from January 1 to 22, 2: from January 23 to February 5, 3: from February 6 to 18, 4: from February 19 to March 11, 5: from March 12 to 26, 6: from March 27 to April 14, 7: from April 15 to May 17, 8: from May 18 to 23, 9: from May 24 to June 11, 10: from June 12 to 25, 11: from June 26 to July 15, 12: from July 16 to 28, 13: from July 29 to August 23, 14: from August 24 to September 7, 15: from September 8 to 18, 16: from September 19 to October 8, 17: from October 9 to November 2, 18: from November 3 to 15, 19: from November 16 to 23, 20: from November 24 to 30, 21: from December 1 to 18, 22: from December 19 to 31

The top five values of the transmission relative rate, as indicated in Fig. 14 were *β*_61_ (mean: 5.76 × 10^-8^, 95% *CI*: 3.96 × 10^− 8^ to 7.56 × 10^-8^), *β*_31_ (mean: 5.32 × 10^-8^, 95% *CI*: 3.91 × 10^-8^ to 6.74 × 10^-8^), *β*_34_ (mean: 4.01 × 10^-8^, 95% *CI*: 3.19 × 10^-8^ to 4.84 × 10^-8^), *β*_62_ (mean: 7.52 × 10^-9^, 95% *CI*: 3.23 × 10^-9^ to 1.18 × 10^-8^) and *β*_64_ (mean: 6.04 × 10^-9^, 95% *CI*: 2.41 × 10^-9^ to 9.67 × 10^-9^).

### Sensitivity analysis

**Fig. 15:**
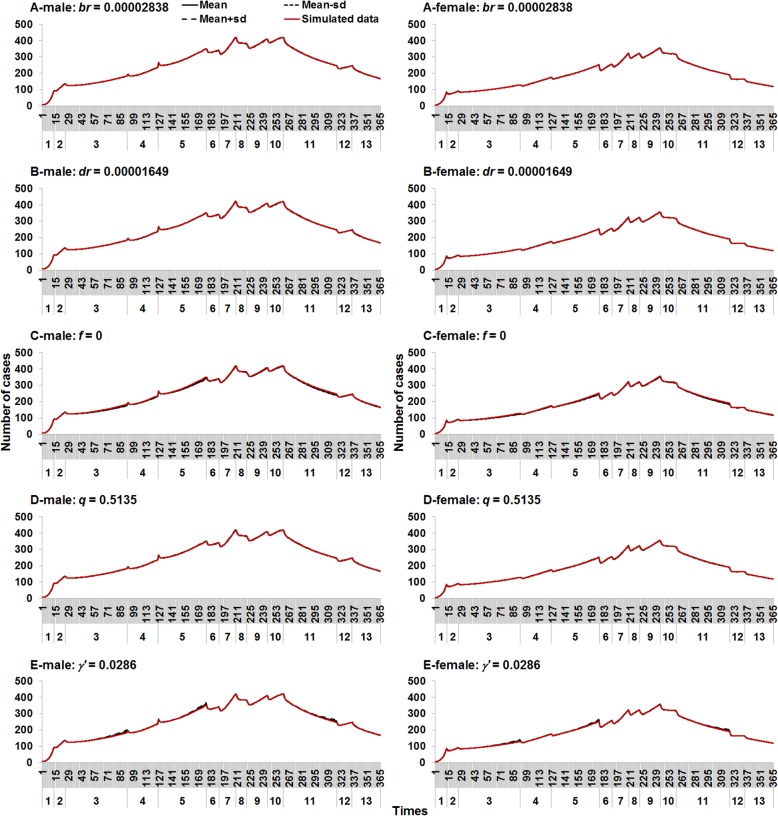
The sensitivity analysis of parameter *br*, *dr*, *f*, *q* and *γ’.* A-male: *br* = 0.00002838; B-male: *dr* = 0.00001649; C-male: *f* = 0; D-male: *q* = 0.5135; E-male: *γ’* = 0.0286; A-female: *br* = 0.00002838; B-female: *dr* = 0.00001649; C-female: *f* = 0; D-female: *q* = 0.5135; E-female: *γ’* = 0.0286

**Fig. 16:**
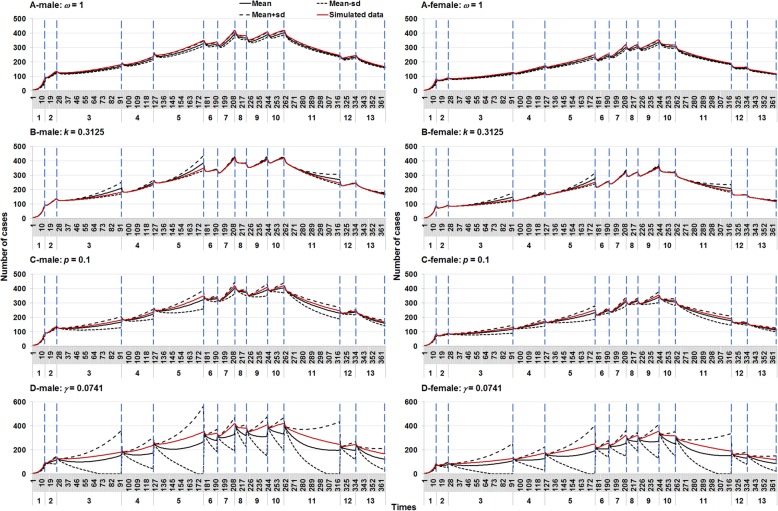
The sensitivity analysis of parameter *ω*, *k*, *p* and *γ.* A-male: *ω* = 1; B-male: *k* = 0.3125; C-male: *p* = 0.1; D-male: *γ* = 0.0741; A-female: *ω* = 1; B-female: *k* = 0.3125; C-female: *p* = 0.1; D-female: *γ* = 0.0741

Based on the 1000 times that the model ran, the model was not sensitive to the parameters *br*, *dr*, *f*, *q* and *γ’*. The number of cases set were the same for the mean, mean – standard deviation (*SD*) and mean + *SD* values (Fig. 15). Our model was slight sensitive with parameters *ω*, *k* and *p* (Fig. 16a,b,c). Meanwhile, high sensitivity to parameter *γ* (0.0741) was demonstrated, as illustrated in Fig. 16d.

## Discussion

Several mathematical models (such as the time-series Susceptible–Infectious–Recovered and SEIARW) have been established to determine the dynamics of shigellosis [17, 35]. However, our study is the first to clarify the transmission of shigellosis between both genders globally. In this study, we used the SEIAR model to study the transmission of the water/food-borne infectious disease and explored the transmission routes in the different sex-age groups further. The results provide guiding significance for controlling the prevalence of shigellosis.

### Model validity

According to *R*^*2*^ of the linear regression, the SEIAR model exhibited a high goodness of fit with the reported data in the different genders. Moreover, it was consistent with the results of previous research [17], suggesting that the model was suitable for this study. According to the results of the sensitivity analysis, the model was more sensitive to parameter *γ*. Therefore, the results would be more reliable if *γ* was collected from real data, instead of from the literature.

## Epidemiological characteristics

In recent years, although the incidence of shigellosis exhibited a decreasing trend in China [6, 26, 36], relatively high levels still occurred in Hubei Province from 2005 to 2017. Different incidences of shigellosis cases in males and females were observed by the descriptive epidemiology [37, 38]. However, few clarifications of the causes of this difference and the transmission features have been provided. A study indicated that there were more cases in males than in females (the male-to-female ratio was 1.3:1), which is consistent with our results in the descriptive epidemiology [39].

The transmission pattern of shigellosis has shifted from water/food-to-person to person-to-person, with high risk groups being particularly men who have sex with other men (MSM) in developed countries [1]. Meanwhile, numerous studies have reported that the incidence in males is higher than that in female [6–8]. Does this mean that the transmissibility of shigellosis among males is stronger than that among females? The SEIAR model was developed to verify this hypothesis. However, we obtained the number of cases in five hypotheses using “knock-out” simulation. When *β*_*fm*_ = 0, the number of cases decreased the most in both genders, which means that female-to-male transmission contributed significantly during the transmission. Therefore, it is important to isolate and treat female cases as well as to strengthen personal health.

### Transmissibility of shigellosis in different genders

In this study, we modelled the reported data from two cities in Hubei Province. The results of the “knock-out” simulation demonstrated that the decreasing trend of Wuhan City was similar to that of Yichang City, but both exhibited a certain disparity compared to the results of Hubei Province. According to Fig. 9, there were differences in the cases reported from Wuhan City and Yichang City for 2010. Both cities exhibited similar ascending and descending trends during each time for the same gender, but the results differed from those of Hubei Province. This could be related to the proportion of male and female cases reported daily. Regional differences may not be the main influential factor for the incidences in terms of gender.

Compared to HIV which exhibits different transmissibility in different genders, shigellosis is not particularly highly contagious in the different genders [40]. Our results demonstrated that the mean values of the transmission parameters among males and females, from male to female, and from female to male are differed, with the following order: *β*_*fm*_ > *β*_*mm*_ > *β*_*mf*_ > *β*_*ff*_. The median values of the *SAR* exhibited the following order: *SAR*_*fm*_ > *SAR*_*mf*_ > *SAR*_*mm*_ > *SAR*_*ff*_. Because a model of the total population in Hubei was constructed, the value of *SAR* was small and within the neighborhood of zero. However, this did not affect the quantification of the transmissibility of shigellosis. A previous study indicated a high incidence in MSM in developed countries owing to unprotected sex and oro-anal contact [1]. However, the proportion of MSM in China is not large. This finding may be related to the fact that the contact rate between males and females, such as kissing, embracing, and shaking hands, is higher than within genders. The results indicate that the most significant transmission route is from female to male. Superior hygiene behaviours may be responsible for the lower female than male incidences. The greatest reason that males are more susceptible than females may be related to superior lifestyle habits, such as hand washing, in female individuals than in males. Moreover, females generally carry out more tasks such as cooking in the home. This finding suggests the importance of emphasizing the importance of washing hands before cooking for females.

The results of this study are consistent with those of most research [41, 42], which have indicated a heavy disease burden in children under 6 years. There is no doubt that children have a relatively high susceptibility compared to other ages. Furthermore, it is apparent that children often exhibit poor habits such as not washing their hands after using the toilet or before meals. Our results demonstrate that the main transmission route is from the elderly to children. There is a custom in China whereby young parents leave their children in the grandparents’ care. This suggests that the most important intervention may be the need to cut off transmission from the elderly. According to the epidemic characteristics of bacterial dysentery, control measures could be implemented in terms of following aspects:

- Focus on females cooking in the home and grandparents caring for grandchildren, such as advocating hand washing.
- Encourage effective hygiene habits to reduce the susceptibility of male individuals and children.
- Reduce the frequency of social behaviour such as kissing, embracing and shaking hands.

### Limitations

Several influential factors contributed to the year 2010 being considered for estimating the transmission features in the different age groups. It is possible that the transmission would vary according to changes in human behaviour. Thus, further research is required to explore the transmission characteristics of Hubei Province.

Numerous studies have indicated that *Shigella* consists of four species, namely *dysenteriae*, *boydii*, *flexneri*, and *sonnei*, among which the final two are the most common in low- and middle-income countries [36, 43, 44]. In our study, the dataset was obtained from routine infectious disease surveillance of the CDC in Hubei Province with no reported information regarding the *Shigella* species. We believe that it is highly necessary to estimate the transmissibility in different *Shigella* species. Additional data for the different species will need to be collected for analysis.

The results have been affected given that we supposed that *β*_*w*_ = 0 in the SEIAR model and ignored environmental factors (such as water and food). Moreover, owing to the limited availability of data, sociological components (for example, occupations, and cultural and societal backgrounds) were not considered in the model. Additional data relating to sociological factors need to be collected for analysis. Finally, the parameters of the SEIAR model were obtained from relevant references and the Hubei Statistical Yearbook, and not from a first-hand data, which had an impact on the accuracy of our model.

## Conclusions

In Hubei Province, the incidence of shigellosis in males is higher than that in females. The transmissibility between the genders is higher than that within the genders, particularly female-to-male transmission. The main transmission route in children (age ≤ 5 years) is transmission from the elderly (age ≥ 60 years). Therefore, the greatest interventions should be applied in females and the elderly.

## Supplementary information

**Additional file 1 **The contribution of *β*_*w*_ in SEIARW model.

**Additional file 2.** Sex-age based SEIAR model.

## Data Availability

Extra data is available by emailing to Dr. Qi Chen (317342267@qq.com) on reasonable request.

## Abbreviations

CDC: Center for Disease Control and Prevention
SEIARW: Susceptible–Exposed–Infectious/Asymptomatic–Recovered–Water/Food
SEIAR: Susceptible–Exposed–Infectious/Asymptomatic–Recovered
*SAR*: Secondary attack rate
*CI*: Confidence interval
*SD*: Standard deviation
MSM: Men who have sex with other men
