# Supplementary material for: Relative transmissibility of shigellosis among male and female individuals: a modeling study in Hubei Province, China
Source: Infect Dis Poverty. 2020 Apr 17;9:39. doi: 10.1186/s40249-020-00654-x (PMC7162736; doi:10.1186/s40249-020-00654-x)
Supplement: Supplementary file 1 — Additional file 1 The contribution of βw in SEIARW model. [file 40249_2020_654_MOESM1_ESM.docx]

**Additional file 1**

**The contribution of *β_w_* in SEIARW model**

**Table 1. Estimated values of *b* and *b_w_* from 2005 to 2017 in Hubei Province, China**

| Year | *b* | 95% *CI* of *b* | | *b_w_* | 95% *CI* of *b_w_* | |
| --- | --- | --- | --- | --- | --- | --- |
|  | Mean | Lower bound | Upper bound | Mean | Lower bound | Upper bound |
| 2005 | 0.0816 | 0.0623 | 0.1009 | 8.5900 × 10^-9^ | -4.0432 × 10^-9^ | 2.1223 × 10^-8^ |
| 2006 | 0.1054 | 0.0857 | 0.1250 | 5.5557 × 10^-9^ | 2.8943 × 10^-9^ | 8.2171 × 10^-9^ |
| 2007 | 0.0881 | 0.0708 | 0.1055 | 1.9438 × 10^-11^ | 1.5076 × 10^-11^ | 2.3799 × 10^-11^ |
| 2008 | 0.0937 | 0.0753 | 0.1121 | 3.5762 × 10^-12^ | 1.7830 × 10^-12^ | 5.3694 × 10^-12^ |
| 2009 | 0.0917 | 0.0724 | 0.1109 | 3.4175 × 10^-13^ | 2.9759 × 10^-13^ | 3.8591 × 10^-13^ |
| 2010 | 0.0792 | 0.0569 | 0.1014 | 3.3700 × 10^-13^ | 1.5747 × 10^-13^ | 5.1653 × 10^-13^ |
| 2011 | 0.0840 | 0.0561 | 0.1120 | 2.6340 × 10^-13^ | 8.0393 × 10^-14^ | 4.4641 × 10^-13^ |
| 2012 | 0.0845 | 0.0697 | 0.0993 | 1.4250 × 10^-13^ | 1.1837 × 10^-13^ | 1.6663 × 10^-13^ |
| 2013 | 0.0908 | 0.0619 | 0.1197 | 9.2350 × 10^-14^ | 5.0479 × 10^-14^ | 1.3422 × 10^-13^ |
| 2014 | 0.0905 | 0.0739 | 0.1072 | 1.2351 × 10^-14^ | 5.5676 × 10^-15^ | 1.9135 × 10^-14^ |
| 2015 | 0.0787 | 0.0590 | 0.0983 | 4.6233 × 10^-15^ | 9.4468 × 10^-16^ | 8.3020 × 10^-15^ |
| 2016 | 0.0889 | 0.0556 | 0.1221 | 3.1740 × 10^-15^ | 2.5692 × 10^-15^ | 3.7788 × 10^-15^ |
| 2017 | 0.0816 | 0.0697 | 0.0935 | 2.7180 × 10^-15^ | 1.4383 × 10^-15^ | 3.9977 × 10^-15^ |
| Pooled | 0.0898 | 0.0851 | 0.0946 | 1.1264 × 10^-9^ | 4.1123 × 10^-10^ | 1.8416 × 10^-9^ |

*CI*: confidence interval.


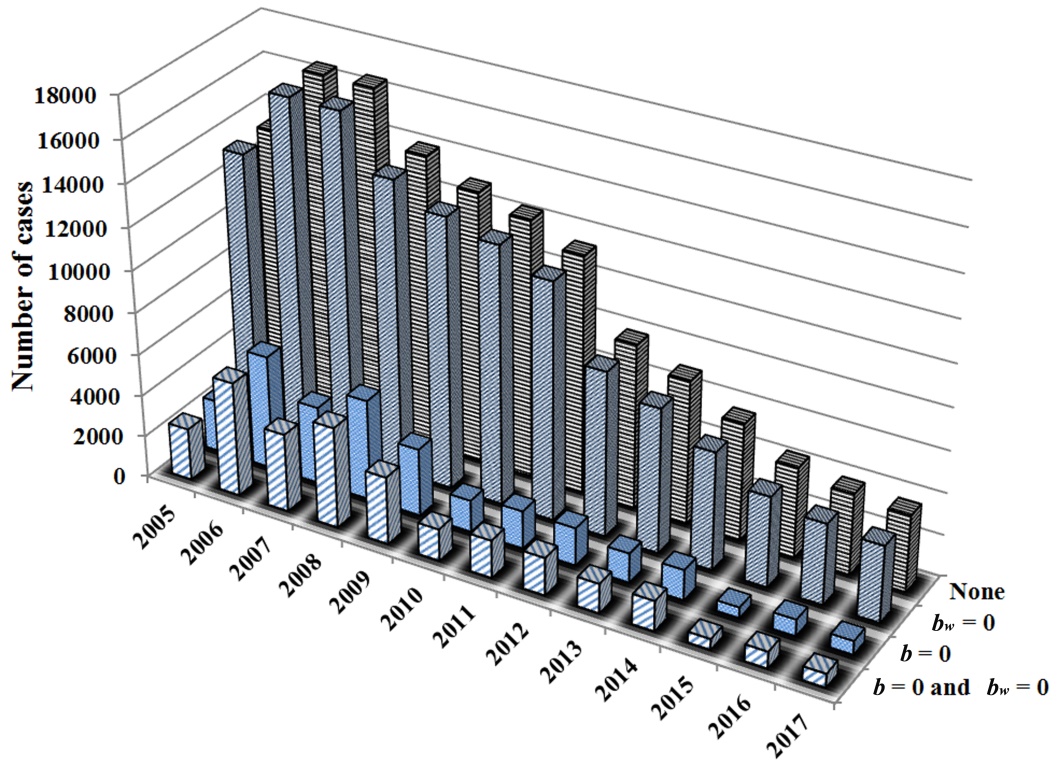


**Figure 1. The “knock-out” simulation of the contribution of *b* and *b_w_* during the transmission (*b* = 0 and *b_w_* = 0; *b* = 0; *b_w_* = 0; and control denoted as “None”; *b*: transmission route from person-to-person; *b_w_*: transmission route from water/food-to-person).**
