# Supplementary material for: Relative transmissibility of shigellosis among male and female individuals: a modeling study in Hubei Province, China
Source: Infect Dis Poverty. 2020 Apr 17;9:39. doi: 10.1186/s40249-020-00654-x (PMC7162736; doi:10.1186/s40249-020-00654-x)
Supplement: Supplementary file 2 — Additional file 2. Sex-age based SEIAR model. [file 40249_2020_654_MOESM2_ESM.docx]

**Additional file 2**

**Sex-age based SEIAR model**

In the model, we divide total individuals into 6 groups (1: male, ≤ 5; 2: male, 6-59; 3: male, ≥ 60; 4: female, ≤ 5; 5: female, 6-59; 6: female, ≥ 60). The equation was expressed as follows:

$$\frac{dS_{1}}{dt}=brNq-\beta_{11}S_{1}\left( I_{1}+kA_{1} \right)-\beta_{21}S_{1}\left( I_{2}+kA_{2} \right)-\beta_{31}S_{1}\left( I_{3}+kA_{3} \right)-\beta_{41}S_{1}\left( I_{4}+kA_{4} \right)-\beta_{51}S_{1}\left( I_{5}+kA_{5} \right)-\beta_{61}S_{1}\left( I_{6}+kA_{6} \right)-drS_{1}$$

$$\frac{dE_{1}}{dt}=\beta_{11}S_{1}\left( I_{1}+kA_{1} \right)+\beta_{21}S_{1}\left( I_{2}+kA_{2} \right)+\beta_{31}S_{1}\left( I_{3}+kA_{3} \right)+\beta_{41}S_{1}\left( I_{4}+kA_{4} \right)+\beta_{51}S_{1}\left( I_{5}+kA_{5} \right)+\beta_{61}S_{1}\left( I_{6}+kA_{6} \right)-\omega E_{1}-drE_{1}$$

$$\frac{dI_{1}}{dt}=\left( 1-p \right)\omega E_{1}-\gamma I_{1}-(dr+f)I_{1}$$

$$\frac{dA_{1}}{dt}=p\omega E_{1}-\gamma^{'}A_{1}-drA_{1}$$

$$\frac{dR_{1}}{dt}={\gamma I}_{1}+{\gamma^{'}A}_{1}-drR_{1}$$

$$\frac{dS_{2}}{dt}=-\beta_{22}S_{2}\left( I_{1}+kA_{1} \right)-\beta_{12}S_{2}\left( I_{1}+kA_{1} \right)-\beta_{32}S_{2}\left( I_{3}+kA_{3} \right)-\beta_{42}S_{4}\left( I_{4}+kA_{4} \right)-\beta_{52}S_{2}\left( I_{5}+kA_{5} \right)-\beta_{62}S_{2}\left( I_{6}+kA_{6} \right)-drS_{2}$$

$$\frac{dE_{2}}{dt}=\beta_{22}S_{2}\left( I_{1}+kA_{1} \right)+\beta_{12}S_{2}\left( I_{1}+kA_{1} \right)+\beta_{32}S_{2}\left( I_{3}+kA_{3} \right)+\beta_{42}S_{4}\left( I_{4}+kA_{4} \right)+\beta_{52}S_{2}\left( I_{5}+kA_{5} \right)+\beta_{62}S_{2}\left( I_{6}+kA_{6} \right)-\omega E_{2}-drE_{2}$$

$$\frac{dI_{2}}{dt}=\left( 1-p \right)\omega E_{2}-\gamma I_{2}-(dr+f)I_{2}$$

$$\frac{dA_{2}}{dt}=p\omega E_{2}-\gamma^{'}A_{2}-drA_{2}$$

$$\frac{dR_{2}}{dt}={\gamma I}_{2}+{\gamma^{'}A}_{2}-drR_{2}$$

$$\frac{dS_{3}}{dt}=-\beta_{33}S_{3}\left( I_{3}+kA_{3} \right)-\beta_{13}S_{3}\left( I_{1}+kA_{1} \right)-\beta_{23}S_{3}\left( I_{2}+kA_{2} \right)-\beta_{43}S_{3}\left( I_{4}+kA_{4} \right)-\beta_{53}S_{3}\left( I_{5}+kA_{5} \right)-\beta_{63}S_{3}\left( I_{6}+kA_{6} \right)-drS_{3}$$

$$\frac{dE_{3}}{dt}=\beta_{33}S_{3}\left( I_{3}+kA_{3} \right)-\beta_{13}S_{3}\left( I_{1}+kA_{1} \right)-\beta_{23}S_{3}\left( I_{2}+kA_{2} \right)-\beta_{43}S_{3}\left( I_{4}+kA_{4} \right)-\beta_{53}S_{3}\left( I_{5}+kA_{5} \right)-\beta_{63}S_{3}\left( I_{6}+kA_{6} \right)-\omega E_{3}-drE_{3}$$

$$\frac{dI_{3}}{dt}=\left( 1-p \right)\omega E_{3}-\gamma I_{3}-(dr+f)I_{3}$$

$$\frac{dA_{3}}{dt}=p\omega E_{3}-\gamma^{'}A_{3}-drA_{3}$$

$$\frac{dR_{3}}{dt}={\gamma I}_{3}+{\gamma^{'}A}_{3}-drR_{3}$$

$$\frac{dS_{4}}{dt}=brN(1-q)-\beta_{44}S_{4}\left( I_{4}+kA_{4} \right)-\beta_{14}S_{4}\left( I_{1}+kA_{1} \right)-\beta_{24}S_{4}\left( I_{2}+kA_{2} \right)-\beta_{34}S_{4}\left( I_{3}+kA_{3} \right)-\beta_{54}S_{4}\left( I_{5}+kA_{5} \right)-\beta_{64}S_{4}\left( I_{6}+kA_{6} \right)-drS_{4}$$

$$\frac{dE_{4}}{dt}=\beta_{44}S_{4}\left( I_{4}+kA_{4} \right)+\beta_{14}S_{4}\left( I_{1}+kA_{1} \right)+\beta_{24}S_{4}\left( I_{2}+kA_{2} \right)+\beta_{34}S_{4}\left( I_{3}+kA_{3} \right)-\beta_{54}S_{4}\left( I_{5}+kA_{5} \right)+\beta_{64}S_{4}\left( I_{6}+kA_{6} \right)-\omega E_{4}-drE_{4}$$

$$\frac{dI_{4}}{dt}=\left( 1-p \right)\omega E_{4}-\gamma I_{4}-(dr+f)I_{4}$$

$$\frac{dA_{4}}{dt}=p\omega E_{4}-\gamma^{'}A_{4}-drA_{4}$$

$$\frac{dR_{4}}{dt}={\gamma I}_{4}+{\gamma^{'}A}_{4}-drR_{4}$$

$$\frac{dS_{5}}{dt}=-\beta_{55}S_{5}\left( I_{5}+kA_{5} \right)-\beta_{15}S_{5}\left( I_{1}+kA_{1} \right)-\beta_{25}S_{5}\left( I_{2}+kA_{2} \right)-\beta_{35}S_{5}\left( I_{3}+kA_{3} \right)-\beta_{45}S_{5}\left( I_{4}+kA_{4} \right)-\beta_{65}S_{5}\left( I_{6}+kA_{6} \right)-drS_{5}$$

$$\frac{dE_{5}}{dt}=\beta_{55}S_{5}\left( I_{5}+kA_{5} \right)+\beta_{15}S_{5}\left( I_{1}+kA_{1} \right)+\beta_{25}S_{5}\left( I_{2}+kA_{2} \right)+\beta_{35}S_{5}\left( I_{3}+kA_{3} \right)+\beta_{45}S_{5}\left( I_{4}+kA_{4} \right)+\beta_{65}S_{5}\left( I_{6}+kA_{6} \right)-\omega E_{5}-drE_{5}$$

$$\frac{dI_{5}}{dt}=\left( 1-p \right)\omega E_{5}-\gamma I_{5}-(dr+f)I_{5}$$

$$\frac{dA_{5}}{dt}=p\omega E_{5}-\gamma^{'}A_{5}-drA_{5}$$

$$\frac{dR_{5}}{dt}={\gamma I}_{5}+{\gamma^{'}A}_{5}-drR_{5}$$

$$\frac{dS_{6}}{dt}=-\beta_{66}S_{6}\left( I_{6}+kA_{6} \right)-\beta_{16}S_{6}\left( I_{1}+kA_{1} \right)-\beta_{26}S_{6}\left( I_{2}+kA_{2} \right)-\beta_{36}S_{6}\left( I_{3}+kA_{3} \right)-\beta_{46}S_{6}\left( I_{4}+kA_{4} \right)-\beta_{56}S_{6}\left( I_{5}+kA_{5} \right)-drS_{6}$$

$$\frac{dE_{6}}{dt}=-\beta_{66}S_{6}\left( I_{6}+kA_{6} \right)-\beta_{16}S_{6}\left( I_{1}+kA_{1} \right)-\beta_{26}S_{6}\left( I_{2}+kA_{2} \right)-\beta_{36}S_{6}\left( I_{3}+kA_{3} \right)-\beta_{46}S_{6}\left( I_{4}+kA_{4} \right)-\beta_{56}S_{6}\left( I_{5}+kA_{5} \right)-\omega E_{6}-drE_{6}$$

$$\frac{dI_{6}}{dt}=\left( 1-p \right)\omega E_{6}-\gamma I_{6}-(dr+f)I_{6}$$

$$\frac{dA_{6}}{dt}=p\omega E_{6}-\gamma^{'}A_{6}-drA_{6}$$

$$\frac{dR_{6}}{dt}={\gamma I}_{6}+{\gamma^{'}A}_{6}-drR_{6}$$

$$N=S_{1}+S_{2}+S_{3}+S_{4}+S_{5}+S_{6}+E_{1}+E_{2}+E_{3}+E_{4}+E_{5}+E_{6}+I_{1}+I_{2}+I_{3}+I_{4}+I_{5}+I_{6}+A_{1}+A_{2}+A_{3}+A_{4}+A_{5}+A_{6}+R_{1}+R_{2}+R_{3}+R_{4}+R_{5}+R_{6}$$
